# FDM 3D Printing and Properties of WF/PBAT/PLA Composites

**DOI:** 10.3390/molecules29215087

**Published:** 2024-10-28

**Authors:** Mengya Li, Wen Lei, Wangwang Yu

**Affiliations:** 1 College of Science, Nanjing Forestry University, Nanjing 210037, China; 2 School of Mechanical Engineering, Nanjing Vocational University of Industry Technology, Nanjing 210023, China

**Keywords:** PBAT/PLA, wood flour, composite, FDM 3D printing, cost

## Abstract

Fused deposition molding (FDM) is a commonly used 3D printing method, and polylactic acid (PLA) has become one of the most important raw materials for this technology due to its excellent warping resistance. However, its mechanical properties are insufficient. Polybutylene adipate terephthalate (PBAT) is characterized by high toughness and low rigidity, which can complement the performance of PLA. The biodegradable polymers produced by blending the two have thus been used to replace petroleum-based plastics in recent years, but the high cost of the blends has limited their wide applications. Introducing plant fibers into the blends can not only maintain biodegradability and improve the overall performance of the plastics but also reduce their costs greatly. In this study, the PBAT/PLA blends with a mass ratio of 70/30 were selected and mixed with wood flour (WF) to prepare ternary composites using a FDM 3D printing technique. The effects of WF dosage on the mechanical properties, thermal properties, surface wettability, and melt flowability of the composites were investigated. The results showed that the proper amount of WF could improve the tensile and flexural moduli of the composites, as well as the crystallinity and hydrophobicity of the printed specimens increased with the content of WF, while the melt flow rate decreased gradually. Compared to PBAT/PLA blends, WF/PBAT/PLA composites are less costly, and the composite containing 20 wt.% WF has the best comprehensive performance, showing great potential as raw material for FDM 3D printing.

## 1. Introduction

Three-dimensional printing, also known as additive manufacturing (AM), is an advanced manufacturing technology that forms parts by printing successive layers of material formed on top of each other [1]. It can accomplish designs with higher flexibility and complexity than traditional manufacturing techniques [2], and is currently used in many fields such as energy and environment, aerospace, automotive manufacturing, biomedicine, and smart homes [3,4].

Fused deposition modeling (FDM) is an advanced 3D printing technology for manufacturing plastic products, and this technology can be widely used in aerospace, medical, and automotive industries because of its low cost, simplicity of operation, high accuracy of prototyping, and material adaptability, as well as the ability to produce complex components [5,6]. During the printing process, thermoplastic filaments are extruded through heated nozzles and deposited layer-by-layer to build structures [7]. The raw materials used in FDM are mostly thermoplastic wires, such as polylactic acid (PLA) [8,9,10], acrylonitrile butadiene styrene (ABS) [8,11], polycarbonate (PC) [12,13], and polysulfone (PSU) [14]. Among them, PLA is the most attractive due to its biodegradability and environmentally friendly properties [15], in addition to the fact that it has low shrinkage force, is less prone to warping, and is easy to print [11].

However, PLA has the shortcomings of low toughness, high brittleness, poor thermal stability, and low crystallinity, which limit its wide application in 3D printing [16]. Therefore, PLA is often modified by blending it with other biodegradable polymers, such as polybutylene succinate (PBS) [17], polycaprolactone (PCL) [18], or polyglycolic acid (PGA) [19], to improve its toughness and expand its application for 3D printing. Polybutylene terephthalate adipate (PBAT), which has a high toughness and a low rigidity, is highly synergistic with PLA as it has complementary properties to biodegradable polymers [20]. Blending PLA with PBAT can not only maintain the biodegradability of the material but also play the role of toughening. Even so, the high price of PLA would hardly be reduced after being blended with PBAT because of the same high price of PBAT, which is a major barrier to the commercialization of PBAT/PLA blend-based products and also problematizes their wider applications in FDM 3D printing just like that of pure PLA. Therefore, cost reduction is still of great significance for the wide adoption of the PBAT/PLA blends in FDM 3D printing.

In recent years, more and more researchers have focused on green composites by adding natural fibers to biopolymers in order to enhance the polymer matrix material and reduce the cost. For example, Yang et al. [21] produced flax fiber/PLA prepreg filaments through 3D printing and found that the tensile strength and tensile modulus of the samples were increased by 30.0% and 57.1%, respectively, from those of PLA. Bundit et al. [22] prepared PLA/PBS/coconut fiber biodegradable composites for 3D printing; it was found that the best overall performance of the composite was achieved when coconut fiber was added at 3.0 wt.%, and its tensile and impact strengths were improved by 47.1% and 7.4%, respectively, over PLA/PBS. Ehsan et al. [23] explored the mechanical properties of 3D printed WF/PLA composites; the results showed that the flexural modulus and stiffness of the samples were increased after the addition of wood flour, and the flexural strength and flexural destructive strain of wood flour (WF)/PLA were increased by 39.0% and 21.0%, respectively, when 5.0 wt.% WF was used. Dönitz et al. [24] performed additive manufacturing of short hemp fiber-reinforced PBS (SF-PBS) and long hemp fiber-reinforced PBS (LF-PBS) using the fused filament fabrication (FFF) technique. The mechanical properties of the two were comparatively investigated. The stiffness of the polymer was affected to almost the same extent for SF-PBS and LF-PBS, with an increase of about 59.0% and 63.0%, respectively, compared to PBS. The strength was decreased by about 23.0% for SF-PBA, much smaller than that for LF-PBS, which was 32.0%.

As the by-product of the wood processing industry, WF is often used as the reinforcing filler for its low cost, low density, good biodegradability, and low mechanical loss [25]. Blending WF in biopolymer can reduce the material cost, impart woodiness to the fabricated parts, and improve the stability of specimen molding [26]. Thus, it has been reported to be blended with PBAT/PLA blends by some traditional processing methods; for example, Chaiwutthinan et al. [27] added WF to PBAT/PLA blends and prepared composites using injection molding. Young’s modulus, the flexural modulus, and the tensile strength increased with the increase of WF content, and WF was beneficial to the water absorption of the composites, which increased from 0.6% to about 66.2% at 30.0 wt.% WF content. This increase could effectively promote the biodegradation of the composites. Dou et al. [28] used an injection molding process to produce sycamore bark powder (PF)/PBAT/PLA composites. The results showed that the tensile modulus of the composites was increased by about 22.9% when 40.0 wt.% PF was incorporated, and the flexural modulus reached 4250.0 MPa. Xu et al. [29] modified WF with coupling agent KH550 and investigated the changes in the properties of melt-blended and injection-molded WF/PBAT/PLA biodegradable composites before and after the modification of WF. Compared with the unmodified ones, the composites with 2.0 wt.% KH550 had a closer bond between the wood flour and the matrix, and the tensile and flexural strengths of the materials were increased by about 7.0% and 4.3%, respectively.

Even so, the WF/PBAT/PLA biocomposites were hardly reported to be used in FDM 3D printing technology. For making full use of the advantages of this composite and enriching the feedstocks for FDM 3D printing, in this study, wood flour with a content of 0–50 wt.% was blended with PBAT/PLA to prepare WF/PBAT/PLA composites using FDM 3D printing technology. The microstructure of the composites was analyzed with scanning electron microscopy, and the effects of the WF dosage on the mechanical properties, thermal properties, melt fluidity, and surface wettability of the printed specimens were investigated. Meanwhile, the cost of the composites was compared with that of the blend. This work aimed to find a novel feedstock for FDM 3D printing technology, which had the best comprehensive performance and a much lower cost than the PBAT/PLA blend.

## 2. Materials and Methods

### 2.1. Materials

PLA pellets (3052D, American Nature Works Co, Minnetonka, MN, USA) were purchased from Suzhou Benfuzhong Plastics Import & Export Co., Ltd. (Suzhou, China); PBAT pellets, industrial grade, were purchased from Xinjiang Blue Ridge Tunhe Science Co., Ltd. (Cahngji, China); and wood flour (WF) was purchased from Nanjing Dayuan Eco-Construction Group Co., Ltd. (Nanjing, China) and was sieved through 60-mesh sieve.

### 2.2. Sample Preparation

The WF was dried to constant weight in an oven at 105 °C. Various amounts of WF were weighed and mixed with PBAT/PLA blends whose mass ratio of PBAT to PLA was kept at 3:7, as shown in Table 1. Extrusion was performed with a twin-screw extruder (SHJ-20, Nanjing Jute Machinery Co., Ltd., Nanjing, China), the screw rotating speed was 70 r/min, the temperatures in zones one through six were 155 °C, 160 °C, 165 °C, 175 °C, 160 °C, and 150 °C in order, and the head temperature was 155 °C. The extruded wire was pelletized and passed through a single screw extruder (KS-HXY, Suzhou Huanxinyang Electric Co., Ltd., Suzhou, China) to produce filaments with a diameter of 1.75 ± 0.05 mm; the rotating speed of the screw was 20 r/min and the temperature was 140 °C.

Standard printed samples were prepared by a desktop 3D printer (MOSHUS108; Hangzhou 3D Technology Co., Ltd., Hangzhou, China). The FDM 3D printing parameters are shown in Table 2.

### 2.3. Testing and Characterization

#### 2.3.1. Mechanical Testing

The mechanical properties were tested using a universal testing machine (E44.304, MTS Industrial Systems (China) Co., Ltd., Shenzhen, China) at room temperature, and the tensile and flexural tests were carried out with reference to the standard test methods ASTM D 638-2010 [30] and ASTM D 790-2010 [31], respectively. The tensile and bending velocities were, accordingly, 10 mm/min and 5 mm/min. For each set of experiments, five samples were selected and averaged.

#### 2.3.2. Microscopic Morphology Observation

Gold was sprayed on the cross-sectional surface of the tensile samples, and the section morphology was observed using a field emission scanning electron microscope (SEM) (Hitachi SU 8010, Hitachi Corporation, Tokyo, Japan) at an accelerating voltage of 3 kV and a magnification of 2000.

#### 2.3.3. Thermal Stability Characterization

A thermogravimetric analyzer (TG 209F1, NETZSCH-Gerätebau GmbH, Selb, Germany) was used to test the thermal stability of the materials under nitrogen atmosphere. An amount of 3–5 mg of the sample was heated from 20 °C to 600 °C, with a heating rate of 20 °C/min. The temperature of 95% of the residual mass of the sample (T_95_), the maximum decomposition temperature (T_p_), and the carbon residual rate (W) of the specimen were recorded.

#### 2.3.4. Melting and Crystallization Behavior Analysis

The melting and crystallization properties were carried out using a differential scanning calorimeter (DSC214, NETZSCH-Gerätebau GmbH, Selb, Germany). Weighing 3–5 mg of the sample in a crucible, the temperature was increased from 20 °C to 200 °C at a rate of 10 °C/min under nitrogen atmosphere, and a constant temperature at 200 °C for 5 min was used to eliminate the thermal history, followed by cooling down to 20 °C at a rate of 10 °C/min and then reheating to 200 °C at a rate of 10 °C/min. The crystallinity of PLA was calculated according to Equation (1):

(1) $$x_{c}=\frac{\left|\Delta H_{m}+\Delta H_{cc}\right|}{\omega \Delta H*}$$

where $x_{c}$ represented the crystallinity of the printed sample; ω was the mass fraction of PLA in the printed sample; $\Delta H_{m}$ and $\Delta H_{cc}$ were the enthalpy change of melting (J/g) and the enthalpy change of cold crystallization (J/g) of the printed sample, respectively; and ΔH* was the enthalpy of melting 100% crystalline PLA (93.7 J/g [32]).

#### 2.3.5. Contact Angle Testing

The water contact angle θ was determined at room temperature using a contact angle meter (DSA100, KRÜSS GmbH, Borsteler Chaussee, Germany). An amount of 5 μL of distilled water was dropped on the surface of the printed sample, held for 15 s, and then the contact angle θ was read.

#### 2.3.6. Melt Flow Rate Testing

The melt flow rate (MFR) of the specimens was tested using a melt flow rate tester (ZRZ1452, Meister Industrial Systems, Shenzhen, China), the MFR was measured at 2.16 kg load, and the temperatures were set at 170 °C and 190 °C, respectively.

## 3. Results

### 3.1. Appearance Quality

**Figure 1:**
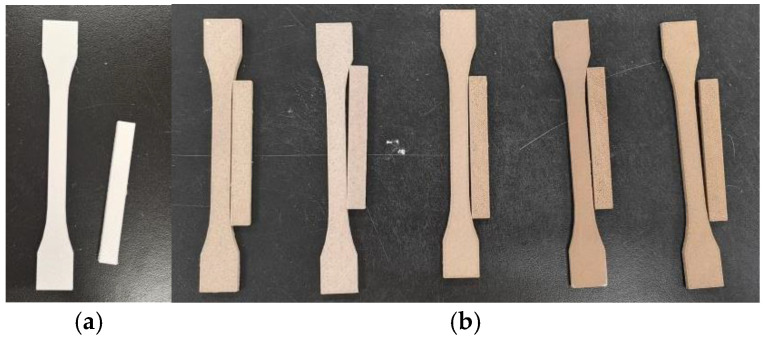
(**a**) Printed PBAT/PLA samples; (**b**) printed WF/PBAT/PLA samples. From left to right: WF-10; WF-20; WF-30; WF-40; WF-50.

The appearances of the printed PBAT/PLA and WF/PBAT/PLA samples are shown in Figure 1.

The printed PBAT/PLA were milky white in color, the adjacent filaments were not tightly bonded, and small gaps could be seen on the surfaces of the printed parts. After the addition of WF, the samples showed a wood color, and the color of the material changed more deeply with the increase of WF content. When printing, WF-10 and WF-20 could be carried out smoothly and evenly, the adjacent filaments could be bonded together tightly, and the surfaces of the printed samples were smooth and free of small gaps. When more WF was used, the smoothness of the filaments decreased and the surfaces of the printed parts became rough.

### 3.2. Mechanical Properties

**Figure 2:**
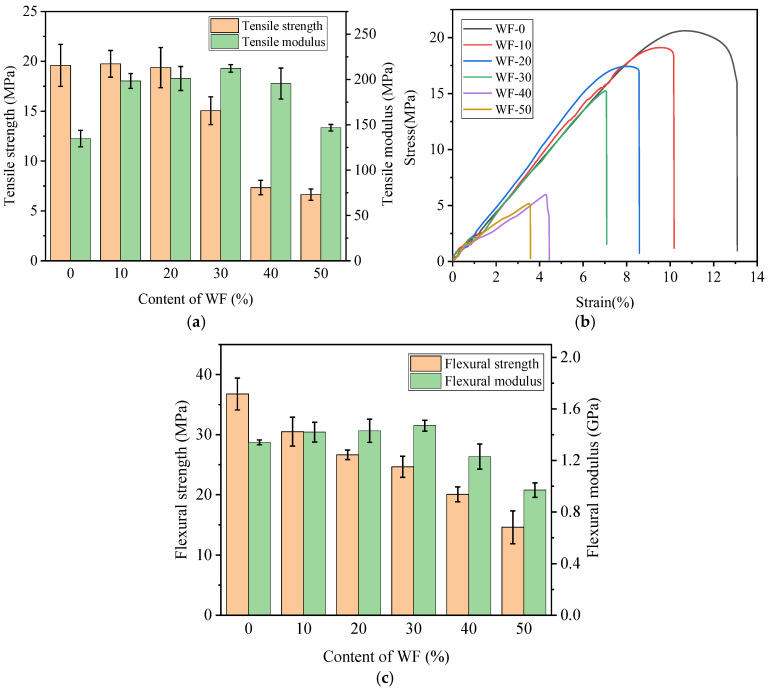
Mechanical properties of WF/PBAT/PLA blends: (**a**) tensile properties; (**b**) stress–strain curves; (**c**) bending properties.

The relationship between the mechanical properties of FDM 3D printed specimens and WF content is shown in Figure 2.

As shown in Figure 2a, the tensile strength and modulus of the printed PBAT/PLA specimens were 20.5 MPa and 139.0 MPa, respectively. When 10 wt.% or 20 wt.% WF was introduced, the tensile strength of the sample remained almost unchanged, and the tensile strength of these two samples changed accordingly to 19.8 MPa and 19.4 MPa. However, when more WF was used, the tensile strength of the sample decreased dramatically; WF-50 only had a tensile strength of 6.6 MPa, which decreased by 67.6% from that of the printed PBAT/PLA sample. For the tensile modulus, it showed a trend of increasing first and then decreasing with the increase of WF content. The tensile moduli of the printed WF-10, WF-20, and WF-30 specimens were 198.4 MPa, 201.1 MPa, and 212.3 MPa, respectively, which were 42.8%, 44.7%, and 52.8% higher than that of the PLA/PBAT blend. When 40 wt.% WF was introduced, and the tensile modulus began to reduce obviously but was still greater than that of the printed PBAT/PLA. The effects of WF addition on the tensile strengths of the samples came from two aspects, one was that wood flour had good rigidity and strength and could play the role of skeleton support in the composite. As a result, the tensile strength of the material would be enhanced when wood flour was used. And the other aspect was that when WF was incorporated into the composite, the poor interfacial compatibility and induced porosity would lead to a significant decrease in the tensile strength. The deterioration of interfacial compatibility could be evidenced in the SEM observations as depicted in subsequent sections. When the blend was complexed with a small amount of WF, the enhancement and deterioration of the tensile strength might be in equilibrium, resulting in an almost unchanged value. When more WF was used, however, more porosities would be produced in the composite, and as a consequence, the strength was worsened more heavily. The increase in modulus could be explained by the effect of the stiffer WF filler on the chain orientation and mobility of the polymer phase, which achieved an effective stress transfer at the fiber–matrix interface [33].

The PBAT/PLA blends had a moderate ductile property and exhibited ductile fracture, as shown in Figure 2b. After the addition of WF, the elongation at break (EAB) of the printed specimens decreased significantly, and the fracture mode changed to be brittle. As the content of wood flour increased, the EAB decreased gradually. The elongation at break of WF-50 was only 4.2%, indicating that the material became more rigid and less tough. This was due to the large amount of cellulose, hemicellulose, and lignin in WF, which had greater brittleness than the polymer matrix. Generally, elongation could be thought of as a reflection of the plasticity of a material [34], and the reduced elongation break indicated that the composites became harder but less ductile compared to the pure PBAT/PLA blend.

The bending performance test results shown in Figure 2c revealed that the bending strength of the printed specimens decreased with the increase of the wood flour filling amount. The flexural strength of WF-0, WF-10, WF-20, WF-30, WF-40, and WF-50 were, accordingly, 36.8 MPa, 30.5 MPa, 25.7 MPa, 24.7 MPa, 20.1 MPa, and 14.6 MPa. The bending modulus increased first and then decreased, but no obvious difference existed among the moduli of WF-10 (1.42 GPa), WF-20 (1.43 GPa), and WF-30 (1.47 GPa). Less WF could be uniformly dispersed in the PBAT/PLA matrix as filler. When the content increased, the dispersion of WF in the matrix became worse and the free volume inside the composite increased; as a result, the bending properties were reduced.

Considering the tensile and flexural properties of the material, it could be found that both the tensile and flexural moduli of WF-20 were a little greater than those of WF-10, while a little smaller than those of WF-30. The tensile and flexural strengths of WF-20 were slightly lower than those of WF-10 while much higher than those of WF-30. Therefore, in terms of mechanical properties, WF-20 should be the most suitable composite for FDM 3D printing.

### 3.3. Microscopic Morphology

**Figure 3:**
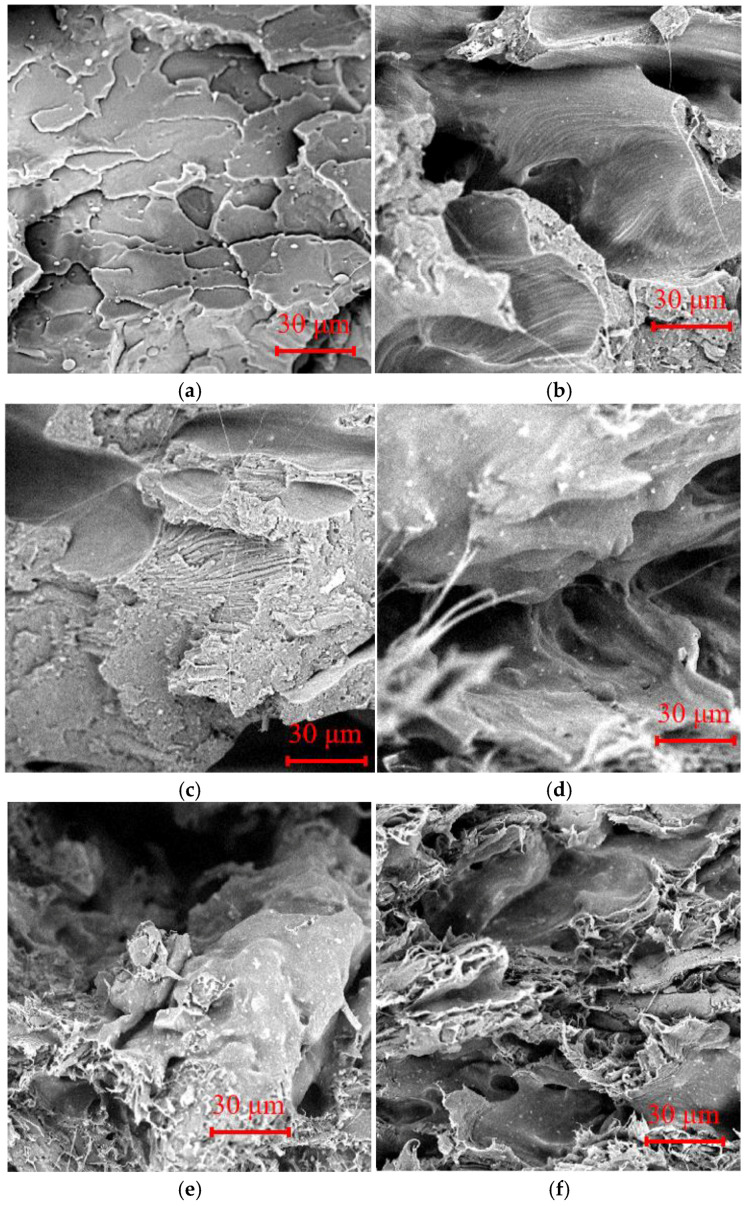
Scanning electron micrographs of WF/PBAT/PLA fracture surfaces (2000×). (**a**) WF-0, (**b**) WF-10, (**c**) WF-20, (**d**) WF-30, (**e**) WF-40, (**f**) WF-50.

Figure 3 shows the cross-sectional morphologies of the printed PBAT/PLA and WF/PBAT/PLA tensile specimens.

In the printed PBAT/PLA (Figure 3a), PLA and PBAT acted as the continuous and dispersed phases, respectively. PBAT was well dispersed in PLA and acted as a toughening agent by inducing a large yield area [20]. For WF/PBAT/PLA containing less WF, i.e., WF-10 and WF-20, the cross-section was still relatively flat, and a small amount of fiber-like structure existed. WF could be uniformly dispersed in the PBAT/PLA matrix (Figure 3b,c). When more WF was introduced, as in Figure 3d–f, the cross-section became rough. The WF detached from the matrix during fracture, leaving a large number of exposed WF fibers on the surface and holes after the WF was pulled out. A large number of hydrophilic hydroxyls in its structure made WF highly polar, leading to its poor interaction with the nonpolar PBAT/PLA polymer. Consequently, the mechanical properties of the samples became poor, as discussed before.

### 3.4. Thermal Stability

**Figure 4:**
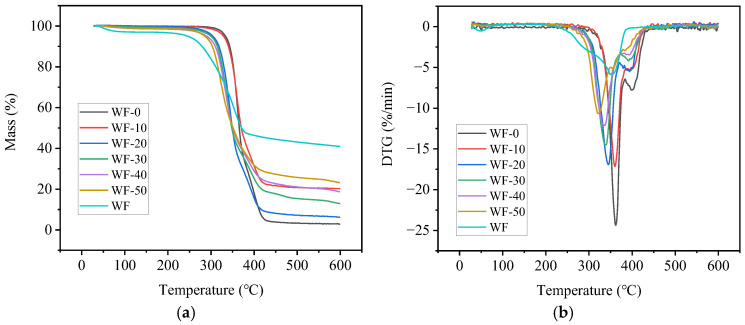
Characterization of thermal stability of WF/PBAT/PLA: (**a**) mass loss curve; (**b**) differential thermogravimetric curve.

Figure 4 shows the TG–DTG curves of the printed PBAT/PLA and WF/PBAT/PLA specimens. The main thermal degradation parameters derived from the figure are listed in Table 3, where T_95_ is the temperature at which 5% of the sample was decomposed, T_p,1_ and T_p,2_ are the peak temperatures of the sample at the fastest decomposition rate, and W is the carbon residual rate of the sample at the time of completion of decomposition.

From Figure 4a, it can be found that each specimen had a tiny mass loss at the temperature below 200 °C due to the evaporation of water adsorbed and small molecules, and much greater mass loss happened between 200 and 500 °C, and all the composites lost mass more than WF, while less than WF-0, indicating that the incorporation of wood flour reduced the thermal stability of the samples. The DTG curves in Figure 4b illustrated that there existed two distinct peaks between 200 and 500 °C for printed PBAT/PLA, implying a significant phase separation between the two polymers. The T_95_ of the blend was 333.3 °C, and the two peaks, i.e., T_p,1_ and T_p,2_, corresponded to 361.9 °C and 400.3 °C, respectively. After being complexed with WF, both T_95_ and T_p,1_ were reduced, and the values decreased gradually with the increasing dosage of WF. However, it could also be observed from Figure 4b that an increased WF would make T_p,2_ become less distinct, implying that the addition of WF was beneficial for the interfacial compatibility between PBAT and PLA. When the carbon residual rate of the specimen at 600 °C was concerned, the residue for PBAT/PLA after decomposition was less than 5%, which implied that the decomposition of PLA and PBAT was almost complete at 600 °C. On the other hand, the carbon residue rate rose significantly with an increased dosage of WF, and the residue of each composite sample was much greater than that of PBAT/PLA. This was attributed to the fact that the carbonized layer formed by the WF at low temperatures [35] prevented the heat from reaching the remaining components. This result suggests that the incorporation of WF inhibited the thermal degradation and retarded the decomposition of PBAT/PLA.

From Table 3, it is evidenced that WF-10 had the greatest T95 and Tp,1 among all the composites, but its carbon residue rate was much smaller than those of the other composites. For WF-20, its T95 and Tp,1 values were both relatively higher than those of WF-30, WF-40, and WF-50, which meant that WF-20 was more thermally stable than the several other mentioned composites. Meanwhile, its W was much greater than that of WF-10, showing that it decomposed much less than the latter. Generally, WF-20 had good thermal stability among all the ternary composites.

### 3.5. Melting and Crystallization Behavior

**Figure 5:**
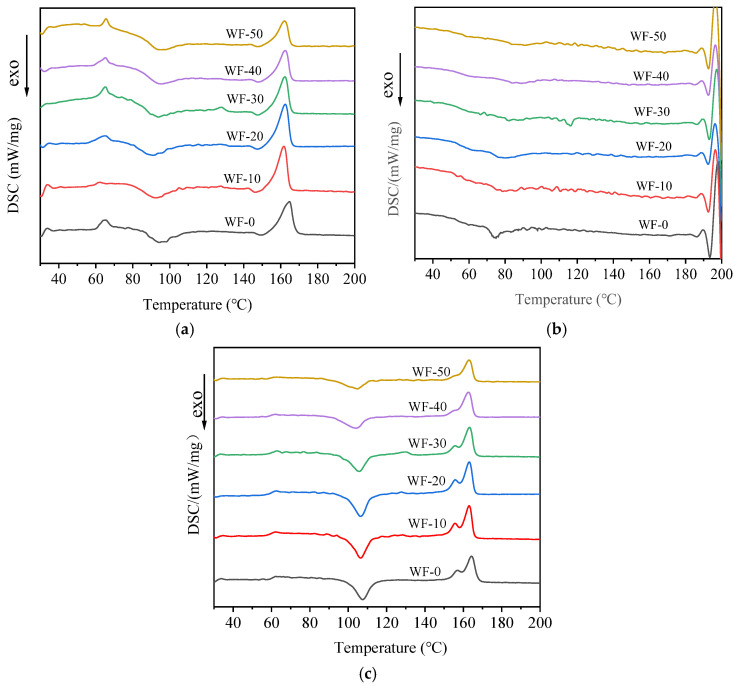
DSC thermograms of WF/PBAT/PLA: (**a**) first heating; (**b**) cooling; (**c**) second heating.

The melting and crystallization behavior of each specimen was characterized by DSC as shown in Figure 5. The thermal history of the printed samples was eliminated by the first heating (Figure 5a); in other words, the effect of various crystalline morphologies arising from polymer processing on the crystallization behavior was removed, and thereby an accurate crystalline behavior of the material itself could be obtained. Glass transition temperature (T_g_), cold crystallization temperature (T_cc_), melting temperature (T_m_), enthalpy of cold crystallization (Δ*H_cc_*), enthalpy of melting (Δ*H_m_*), and degree of crystallinity X_c_ were obtained from the second heating curve (Figure 5c), and the results are tabulated in Table 4.

As can be seen from Table 4, the addition of WF and its content had little effect on the glass transition temperature and melting temperature of the printed samples, whose T_g_ and T_m_ were kept at around 60 °C and 163 °C, respectively. The cold crystallization temperature decreased with the increase of WF content, owing to the heterogeneous nucleation action by WF [36], and thus induced the crystallization of the polymer matrix at a lower temperature. Meanwhile, the presence of wood flour enhanced the chain segment movement ability of the composites and promoted the movement of PLA molecular chains to form an ordered structure, thus leading to a decrease in T_cc_ [37]. The role of the nucleating agent from WF could also be evidenced from the crystallinity results demonstrated in Table 4; the crystallinity of the printed sample increased with the increase of WF content. WF-50 had a crystallinity of 6.3%, 103.5% greater than that of the PBAT/PLA blend. WF is mainly composed of cellulose, hemicellulose, and lignin. The presence of hydroxyl, carboxyl, and ester groups in these structures could form hydrogen bonds with the polymer matrix, thus increasing the crystallinity of PLA. In addition, two melting peaks were observed on the second ramp-up curves for all the composites. The peak at the lower temperature was attributed to the reduced chain alignment and conformational purity of the PLA matrix during melt mixing, leading to the recrystallization of the low-perfect crystals of PLA into α-higher-perfect crystals [38]. The peak at the higher temperature was associated with the melting of perfect crystals formed through the melting recrystallization process [39].

After further investigation of the melting properties of the printed specimens, it was noticed that WF-20 had the greatest ∆H_c_ and ∆*H_m_*, indicating that WF had a perfect crystal structure.

### 3.6. Wettability

**Figure 6:**
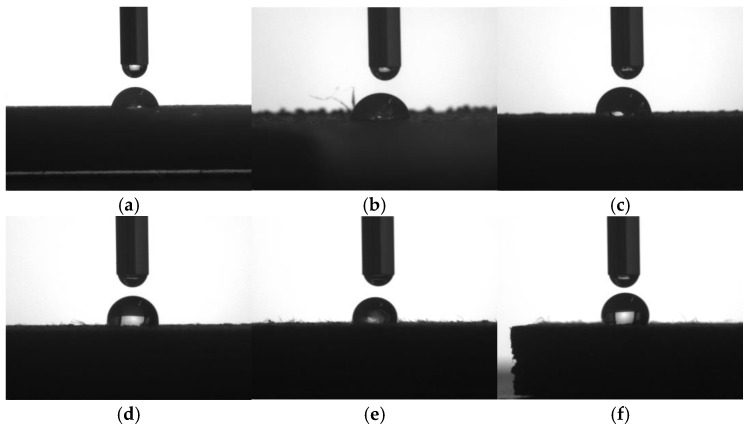
Surface contact angles of samples with different WF contents: (**a**) WF-0, (**b**) WF-10, (**c**) WF-20, (**d**) WF-30, (**e**) WF-40, (**f**) WF-50.

The hydrophilicity of the samples was assessed by testing their water contact angle. The surface contact angle morphology is shown in Figure 6, and the corresponding data are organized in Table 5.

As shown in Table 5, the contact angle of the PBAT/PLA blend was 76.1°, and the contact angle of the composites increased with the increase of WF content; when the WF content was 50 wt.%, the contact angle reached 106.6°, which was an increase of 40.1% over the PBAT/PLA blend. This result was consistent with the study of Ayrilmis et al. [40]. Wood had a much lower surface energy than polymer materials, and the wettability of the materials always increased with increasing surface energy [40]. When more WF was introduced, the surface energy was reduced gradually, leading to poorer wettability, and the contact angle was thus increased. In addition, there existed much wax on the surface of the wood, which was hydrophobic. In this study, WF had not been treated by any method, so its surface should contain some wax. When it was incorporated into the polymer matrix, the hydrophobicity of the printed sample was enhanced; in this situation, the contact angle was also increased.

The transition between hydrophilicity and hydrophobicity of different specimens happened between WF-20 and WF-30: WF-20 behaved hydrophilically just like WF-10 and WF-0, while WF-30 and the other composites behaved hydrophobically. Despite the presence of hydrophilic groups such as hydroxyl groups in WF, its surface often also contains hydrophobic components such as waxes. The combined result of the two leads to a decrease in the hydrophilicity of the ternary composites and a gradual increase in the contact angle. The increased hydrophilicity would make the material take more water, as reported, and more water uptake was helpful for the degradation of a material [27]. So, it could be concluded that the biodegradability of the polymer blend had been little affected when 10 wt.% or 20 wt.% WF was introduced.

### 3.7. Melt Flow Rate

**Figure 7:**
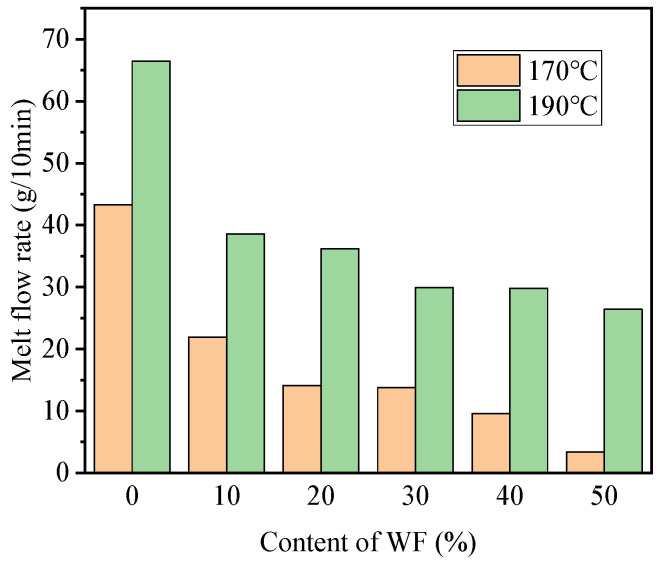
Melt flow rate curves at different WF contents.

FDM is a process of mutual extension bonding between neighboring interfaces of the melt, and the flow properties of the material are related to whether the material can be used for 3D printing. Figure 7 shows the melt flow rate (MFR) of the specimens at different temperatures.

The MFR of PBAT/PLA was 43.3 g/10 min and 66.5 g/10 min at 170 °C and 190 °C, respectively. The addition of 10 wt.% WF resulted in a rapid decrease in the MFR of the composites at both test temperatures. On one hand, the fibers contained a large number of carboxyl groups, which formed a complex rheological system when composited with PLA in the molten state [41]. In addition, as solid particles, WF did not have the flow characteristics of polymer melts. This would hinder the movement of PLA molecular chains to a certain extent, leading to an increase in the melting viscosity of the composites.

At 170 °C, the MFR of WF-20 and WF-30 was smaller than WF-10, but much greater than those of WF-40 and WF-50. At 190 °C, WF-20 had a slightly smaller MFR than WF-10, but was much greater than the other composites. All this means that WF-20 had a moderate flow ability though a little poorer than WF-10. This characteristic made WF-20 easier to be FDM 3D printed.

When comparing the MFR of the same specimen at different temperatures, as expected, the MFR at 190 °C was always greater than that at 170 °C. At higher temperatures, the increased molecular motion of the sample led to an increase in the free space of the PLA macromolecular chains. The intermolecular forces were thus reduced, which in turn increased the fluidity of the material. Moreover, compared to WF-10 and WF-20, the wires of the composites containing more WF were not stacked tightly enough during the printing process.

### 3.8. Cost Analysis

As aforementioned, WF-20 had the best comprehensive mechanical properties, a perfect crystal structure, good thermal stability, and hydrophilicity; meanwhile, it had a moderate MFR and could be printed easily. In summary, it should be an ideal candidate for FDM 3D printing. What was also important was that WF-20 should have a lower material cost than the PBAT/PLA blend.

The current market prices of PLA, PBAT, and wood flour in China are about 480 USD/ton, 525 USD/ton, and 130 USD/ton, respectively. The material cost of the PBAT/PLA would thus be 493.5 USD/ton for WF-20. When 20 wt.% polymer materials were replaced by WF, the material cost was reduced to 420.8 USD/ton, a reduction of about 15% in the cost thus being realized. The reduced material cost made it more competitive in the market.

## 4. Discussions

The effect of WF addition on the comprehensive properties of WF/PBAT/PLA composites prepared using FDM 3D printing technology was investigated in this study, with a focus on improvements in mechanical properties and thermal stability as well as reduction in cost. Compared with previous studies, significant progress has been made by this study, and the material properties of different WF contents were revealed.

As has been demonstrated in previous research, the addition of an appropriate amount of WF could enhance the stiffness and flexural strength of composites. However, many studies were limited to applications with low WF content (typically 5 wt.%–10 wt.%). This study further explored the impact of WF content ranging from 10 wt.% to 50 wt.%, showing that the composite exhibited the best mechanical properties at 20 wt.%. This result is in agreement with previous studies, which indicated that a certain content of WF improved the tensile and flexural modulus of composites. In our study, it was found that the tensile strength and toughness of the materials decreased significantly when the WF content exceeded 30 wt.%. This might be due to the poor interfacial interaction between the matrix and filler. In contrast, many earlier studies did not fully consider the negative impact of high WF content on the interfacial compatibility of the materials.

The effect of WF content on the thermal properties of the materials was also investigated in this study. It was shown in the TGA test that the introduction of WF reduced the temperature of the composite at 5% mass loss (T_95_), while the carbonized layer formed by the WF effectively prevented further thermal degradation at high temperatures. Additionally, the effect of different WF contents on the crystallization temperature and crystallinity of the materials was analyzed with DSC technology in this study. It was pointed out that WF-20 composites had the best crystallization behavior, with a highly ordered crystalline structure. This provided a new direction to improve the dimensional stability of the printed materials.

Furthermore, this study filled the gap in previous research, which focused only on traditional molding processes (e.g., injection molding). This experiment focused on the properties of composites in FDM 3D printing technology. In FDM 3D printing, the stability and precision of the printing process were affected directly by the melt flow rate (MFR) of the material. The impact of WF content on MFR and print quality was thus carried out. It was found that adding a certain amount of WF (with 20 wt.% being optimal) could make the samples maintain better print flowability and shaping quality. The advantages of WF/PBAT/PLA could thus be fully utilized, making the material have great potential in FDM 3D printing.

The incorporation of WF not only improved the mechanical properties of PLA/PBAT blends but also had a wider industrial application prospect. Different from previous studies that focused only on performance optimization, this research also addressed the issue of material cost. This study proposed the feasibility of reducing the cost of expensive PLA/PBAT composites by incorporating WF. It was indicated that the addition of 20 wt.% WF to the PBAT/PLA matrix could reduce the cost by approximately 15%. Combining the excellent properties of WF composites with cost-effectiveness gave the FDM 3D-printed composites significant potential for application in construction materials and other biodegradable plastic alternatives.

In brief, this study demonstrated the great potential of WF/PBAT/PLA composites in FDM 3D printing. It offered a new approach to solving the high cost and insufficient mechanical performance of traditional PLA and PBAT, thereby raising new questions and challenges for future research. First, how to further improve the interfacial compatibility between WF and polymer matrix, especially at high WF content, remains a technical challenge to be solved. Future research could explore chemical modifications (such as the use of coupling agents) or physical treatment methods (such as plasma treatment) to enhance the interfacial bonding between WF and the polymers. Additionally, the effect of the introduction of other biomass materials (e.g., bamboo powder, rice husk) on the properties of PBAT/PLA-based composites could be explored in the future to broaden the application of biodegradable materials.

## 5. Conclusions

In this study, the WF/PBAT/PLA ternary composites were prepared using 3D printing technology, the effects of WF content on the properties of the printed samples were investigated, and the material cost was analyzed. The conclusions can be drawn as follows:

(1) The addition of WF decreased the tensile and flexural strengths of PBAT/PLA, and the tensile and flexural moduli showed a tendency to increase first and then decrease with the increase of WF content. Among them, WF-20 had the best overall mechanical properties, whose tensile strength, tensile modulus, flexural strength, and modulus were 19.4 MPa, 201.1 MPa, 26.7 MPa, and 1.4 GPa, respectively.
(2) Both the T95 and Tp of the printed samples decreased gradually with the increased WF dosage while the char residue rate increased; the thermal stability of the printed specimens worsened when WF was introduced, and the degradation process was retarded. Among all the printed composites, WF-20 had proper thermal stability and decomposition speed.
(3) WF content had little effect on the glass transition temperature and cold crystallization temperature, while it had a positive correlation with the crystallinity of the printed composite samples, WF-20 had the greatest cold crystallization enthalpy and melting enthalpy, showing a perfect crystal structure compared to the composite samples.
(4) As the WF content increased, the hydrophilicity of the composite was enhanced, and the sample turned from surface hydrophilicity to hydrophobicity when the WF content was greater than 20 wt.%.
(5) The MFR of the printed specimens decreased with the increasing dosage of WF, and all the samples had a greater MFR at 190 °C than that at 170 °C.

To sum up, the incorporation and dosage of WF had an effect on the mechanical, thermal, and melting properties of the printed specimens, among which, WF-20 had the best comprehensive properties. Meanwhile, the material cost of WF-20 was reduced by about 15% from that of the PBAT/PLA blend. Taking the material’s properties and cost simultaneously, WF-20 should be an ideal candidate for FDM 3D printing technology.

## Figures and Tables

**Table 1:** Compositions of the samples.

Sample Codes	WF-0	WF-10	WF-20	WF-30	WF-40	WF-50
PLA/wt.%	70	63	56	49	42	35
PBAT/wt.%	30	27	24	21	18	15
WF/wt.%	0	10	20	30	40	50

**Table 2:** Printing parameters.

Parameter	Nozzle Temperature/°C	Platform Temperature/°C	Printing Speed/ mm/s	Layer Thickness/ mm
Value	220	50	50	0.1

**Table 3:** Main thermal degradation parameters of WF/PBAT/PLA.

Sample Code	T_95_/°C	T_p,1_/°C	T_p,2_	W/%
WF-0	333.3	361.9	400.3	4.3
WF-10	329.2	360.1	394.2	9.7
WF-20	304.2	345.2	391.1	22.0
WF-30	299.5	338.8	392.5	24.1
WF-40	290.0	336.2	394.0	28.3
WF-50	285.8	321.3		33.1
WF	245.4	349.2		53.5

**Table 4:** Parameters of WF/PBAT/PLA second heating curve.

Sample Code	T_g_/°C	T_cc_/°C	T_m_/°C	∆*H_cc_* (J/g)	∆*H_m_* (J/g)	X_c_ (%)
WF-0	61.4	107.6	164.2	−17.5	19.5	3.11
WF-10	59.3	106.5	163.0	−16.6	19.3	4.49
WF-20	59.6	106.5	163.1	−19.8	22.3	4.50
WF-30	60.7	105.6	162.9	−16.5	18.8	4.54
WF-40	60.0	104.5	162.7	−11.9	14.4	5.34
WF-50	59.2	103.8	162.8	−9.5	12.5	6.33

**Table 5:** Surface contact angle of WF/PBAT/PLA.

Sample Code	WF-0	WF-10	WF-20	WF-30	WF-40	WF-50
Contact angle/°	76.1 ± 4.7	82.7 ± 4.2	88.5 ± 2.9	96.9 ± 4.6	100.1 ± 5.7	106.6 ± 4.2

## Data Availability

The original contributions presented in this study are included in the article. Further inquiries can be directed to the corresponding author.
